# Longitudinal pathways between childhood BMI, body dissatisfaction, and adolescent depression: an observational study using the UK Millennium Cohort Study

**DOI:** 10.1016/S2215-0366(23)00365-6

**Published:** 2024-01

**Authors:** Emma Blundell, Bianca L De Stavola, Madelaine Davies Kellock, Yvonne Kelly, Gemma Lewis, Anne McMunn, Dasha Nicholls, Praveetha Patalay, Francesca Solmi

**Affiliations:** a Department of Clinical, Educational and Health Psychology, University College London, London, UK; b Institute of Child Health, University College London, London, UK; c Institute of Epidemiology & Health, University College London, London, UK; d Division of Psychiatry, University College London, London, UK; e Centre for Longitudinal Studies and MRC Unit for Lifelong Health & Ageing, University College London, London, UK; f Division of Psychiatry, Imperial College London, London, UK

## Abstract

**Background:**

Globally, more adolescents are having depressive symptoms than in the past. High BMI is a risk factor for depressive symptoms, potentially acting via increased body dissatisfaction. Robust longitudinal evidence of these associations could help to inform preventive interventions, but such evidence remains scarce. We investigated the longitudinal associations between BMI at age 7 years and depressive symptoms at age 14 years (objective 1), BMI at age 7 years and body dissatisfaction at age 11 years (objective 2), and body dissatisfaction at age 11 years and depression at age 14 years (objective 3). We also investigated the extent to which body dissatisfaction mediated the association between BMI and depressive symptoms (objective 4).

**Methods:**

This study used data from the Millennium Cohort Study, a representative longitudinal general population cohort of UK children born between Sept 1, 2000, and Jan 11, 2002. We used univariable and multivariable linear regression models to investigate the associations in objectives 1–3 adjusting for a range of child-level and family-level confounders. For mediation analyses we used non-parametric g-formula (objective 4). We reported stratified results in presence of sex differences. All analyses were based on participants with complete BMI data and imputed confounders and outcomes.

**Findings:**

Our sample included 13 135 participants. Of these, 6624 (50·4%) were male participants and 6511 (49·6%) were female participants; 11 096 (84·4%) were of White ethnicity and 2039 (15·6%) were from a minority ethnic background. At baseline, mean age was 7·2 years (SD 0·25, range 6·3–8·3). In multivariable models, an SD increase in BMI at age 7 years was associated with greater depressive symptoms at age 14 years (estimated regression coefficient [coeff]: 0·30, 95% CI 0·17–0·43) and greater body dissatisfaction at age 11 years (coeff 0·15, 0·12–0·18). Greater body dissatisfaction at age 11 years was associated with higher depressive symptoms at age 14 years (coeff 0·60, 0·52–0·68). All these associations were twice as large in girls as in boys. Body dissatisfaction explained 43% of the association between BMI and depression in girls.

**Interpretation:**

Our findings bear relevance for interventions aimed at reducing weight in childhood and reducing body dissatisfaction. Implementation of evidence-based body image interventions and identification of drivers of weight stigma should be key public health priorities. Interventions aiming to reduce weight in childhood need to avoid increasing body dissatisfaction and should target environmental drivers of weight rather than individuals.

**Funding:**

Wellcome Trust; The Royal Society; Economic and Social Research Council; and the National Institute for Health and Care Research.

## Introduction

The proportion of adolescents with depressive symptoms has increased over the past 20 years,^1^, ^2^ calling for an improved understanding of modifiable risk factors to be targeted by preventative interventions. The average BMI of children and adolescents has also increased over recent decades.^2^ Elevated BMI is a risk factor for depression in adults,^3^ and the scarce longitudinal research in young people also finds that high BMI in adolescence is associated with subsequent increased depressive symptoms, particularly in girls.^4^ However, less is known about the association between BMI in childhood—ie, before the onset of depression—and adolescent depressive symptoms.

Although usually conceptualised as a physical health indicator, BMI is likely to index both physiological (eg, metabolic abnormalities) and environmental processes (eg, weight-based bullying and discrimination). Understanding the mechanisms by which weight status affects adolescent depression could inform effective interventions. Research on the mechanisms linking BMI and depression has largely been conducted in adult populations and has focused on physiological pathways such as metabolic and inflammatory markers, with mixed results.^5^, ^6^ However, metabolic abnormalities are uncommon in childhood and early adolescence,^7^ whereas social environments and peer relationships are important. Psychosocial and environmental mechanisms, compared with biological ones, have been less commonly explored longitudinally.

**Research in context**

**Evidence before this study**

We searched PubMed for any studies published in English investigating the extent to which body dissatisfaction mediates the association between childhood BMI and adolescent depression using the following search terms: “((((((((BMI) OR (obes*)) OR (weight)) AND (((body image) OR (body dissatisfaction)) OR (appearance))) AND ((mediat*) OR (pathway*))) AND ((depress*) OR (mental health))) AND ((mediat*) OR (pathway*))) AND (longitudinal)) AND (((adolescen*) OR (young people)) OR (child*))”. Three previous studies have investigated this question, with contradictory findings. Two of these studies were based in the UK, and one in Canada. Limitations of these studies included small sample sizes and minimal adjustments for key confounders. Only two studies investigated childhood BMI, which is necessary to limit potential for detecting reverse causation, and none used counterfactual-based approaches to causal mediation analysis.

**Added value of this study**

As far as we are aware, this is the largest longitudinal sample investigating the association between childhood BMI and body dissatisfaction and depressive symptoms. It is also the first study investigating the extent to which body dissatisfaction mediates the association between childhood BMI and adolescent depressive symptoms using causal inference approaches to mediation. In a sample of 13 135 UK children and adolescents, after adjusting for a broad range of potential confounders, we found that children with higher BMI at age 7 years had both higher body dissatisfaction at age 11 years and higher depressive symptoms at age 14 years than children with lower BMI at age 7 years. Higher body dissatisfaction at age 11 years was also associated with greater depressive symptom scores at age 14 years. All these associations were twice as large in girls than boys. In girls, body dissatisfaction explained 43% of the association between childhood BMI and adolescent depressive symptoms.

**Implications of all the available evidence**

Body dissatisfaction might be an important modifiable target for preventive interventions aimed at reducing onset of adolescent depressive symptoms, particularly among adolescent girls, who have experienced a large increase in depressive symptoms over the past 2 decades. Development and implementation of evidence-based body image interventions and identification of societal drivers of weight stigma should be key public health priorities. As a large portion of the association between BMI and depressive symptoms can be explained by increased body dissatisfaction, it is also important that any interventions aiming to reduce weight in childhood do not inadvertently increase body dissatisfaction. Interventions aimed at environmental drivers of weight as opposed to individuals could reduce adverse mental health outcomes associated with high BMI.

Body dissatisfaction refers to a person's negative feelings towards their body as a result of a discrepancy between their desired and actual appearance.^8^ In the context of people with higher weights, body dissatisfaction often stems from the internalisation of stigmatising beliefs and societal attitudes towards people with higher weights. As such, body dissatisfaction is a plausible psychosocial mechanism linking high BMI to depression in young people, especially as its prevalence increases between late childhood and early adolescence.^9^ Although body dissatisfaction occurs across the BMI spectrum, it is more common among children and adolescents with higher BMI.^10^ It has been argued that the increased focus on BMI as a key health indicator could have made stigmatising attitudes towards people with higher weights more pronounced in recent decades, and adolescent body dissatisfaction more common across the BMI spectrum.^11^ Only a few longitudinal observational studies have investigated the association between body dissatisfaction and depressive symptoms in children and adolescents, finding evidence of a positive association between the two.^12^, ^13^, ^14^, ^15^ Corroborating this evidence, randomised trials also found that interventions to reduce body dissatisfaction lead to reductions in depressive symptoms.^16^ Nevertheless, most observational studies were based on small samples often not accounting for key confounders, such as pre-existing mental health difficulties or pubertal status, whereas most randomised trials were based on exclusively or predominantly female participants, thus limiting their generalisability to the general population.^16^

To our knowledge, only three longitudinal studies have investigated whether body dissatisfaction mediates the association between childhood BMI and adolescent depression.^17^, ^18^, ^19^ These studies reported contradictory findings and have several limitations. First, only two of these studies used childhood BMI as exposure, the use of which reduces the potential for detection of reverse causation, thus strengthening causal inferences.^18^, ^19^ Second, none of these studies adjusted for potential intermediate confounders, including depressive symptoms.^17^, ^18^, ^19^ Third, by modelling multiple outcomes concomitantly, some of these studies could have adjusted for factors on the causal pathway between exposure and outcome.^18^

There is minimal longitudinal research on the associations between childhood BMI, adolescent body dissatisfaction, and adolescent depressive symptoms, and on the mediating role of body dissatisfaction in the association between childhood BMI and adolescent depressive symptoms. To address these research gaps, we used a longitudinal population-based sample representative of UK children born in the early 2000s, to investigate whether higher BMI at age 7 years was associated with increased (objective 1) depressive symptoms at age 14 years and (objective 2) higher body dissatisfaction at age 11 years; and (objective 3) whether greater body dissatisfaction at age 11 years was associated with increased depressive symptoms at age 14 years. Finally, we investigated (objective 4) the extent to which increased body dissatisfaction mediated the association between higher BMI at age 7 years and greater depressive symptoms at age 14 years.

## Methods

### Study design and participants

We used data from the Millennium Cohort Study, an ongoing UK longitudinal birth cohort study that recruited 18 552 families with a child born between Sept 1, 2000 and Jan 11, 2002 (appendix p 4). Ethics approval to the study was received at each wave, at which point participants also provided written informed consent. To keep timing of confounder measurement consistent across participants, we included children who were in the original cohort sample and had BMI data at age 7 years. In cases of multiple births, we retained one child at random.

### Procedures

At age 14 years, adolescents self-reported depressive symptoms using the 13-item Short Mood and Feelings Questionnaire (sMFQ).^20^ The sMFQ questions are scored on a three-point scale (0=not true, 1=sometimes, 2=true) giving a total score ranging from 0 to 26 (appendix p 4). Higher scores indicate greater depressive symptoms. The sMFQ has been extensively validated, showing measurement invariance across adolescence, sex, and countries.^21^, ^22^ In our sample, the sMFQ had excellent internal consistency (Cronbach's alpha 0·93).

Objective measurements of height and weight were taken at age 7 years by trained interviewers. We calculated BMI (kg/m^2^) and standardised it by age and sex using WHO growth charts as appropriate in this age group.

Body dissatisfaction was measured at age 11 years with the question: “On a scale of 1 to 7 where ‘1’ means completely happy and ‘7’ means not at all happy, how do you feel about the way you look?”. We recoded these values as ranging from 0 to 6, with higher values representing greater dissatisfaction with one's appearance. This item was taken from a battery of questions related to participants’ happiness with different aspects of their lives (eg, school work, family, friends) developed by Chan & Koo.^23^ Although, as far as we are aware, this item has not been validated against body image questionnaires, it has been previously used to capture the construct of body dissatisfaction in several longitudinal cohorts.^17^, ^24^

All models were adjusted for several confounders that we hypothesised could bias the estimated association between exposure and outcomes of interest. We used direct acyclic graphs^25^ to guide our choice of confounders based on evidence from previous literature and clinical observations (appendix p 5). We identified both shared and model-specific confounders in objectives 1–3, which were all included in objective 4.

Shared confounders across models were child's sex, ethnicity, highest parental education and occupation, and equivalised weekly income. We also included maternal age at index child's birth, pre-pregnancy BMI, child's birthweight and gestational age, maternal smoking and drinking habits in pregnancy, and maternal attachment. All these variables were collected when the child was aged 9 months by parent report. We further adjusted models for maternal depressive symptoms measured when the child was aged 3 years and child's emotion dysregulation and mental health difficulties at age 7 years (appendix pp 6–7).

We also controlled our models for child internalising and externalising symptoms reported by parents. In objectives 1, 2, and 4, for which the exposure is BMI at age 7 years, we adjusted for these symptoms at age 7 years, whereas in objective 3, for which body dissatisfaction at age 11 is the exposure, we adjusted for these symptoms at age 11 years. At age 11 years, adolescents were also asked to self-report their mental wellbeing for the first time, so we used that measure in additional sensitivity analyses (appendix pp 6–8). We further adjusted analyses of objective 3 for pubertal development and BMI at age 11 years (appendix pp 6–7).

In mediation models, internalising and externalising symptoms, pubertal status, and BMI at age 11 years were included as intermediate confounders—ie, we considered them as confounders of the association between body dissatisfaction and depressive symptoms, while additionally allowing for BMI at age 7 years as a potential cause of these factors.

### Statistical analysis

All analyses were conducted in Stata 16 and were pre-registered on the Open Science Framework website, with minor deviations reported in the appendix (p 7).

**Figure 1:**
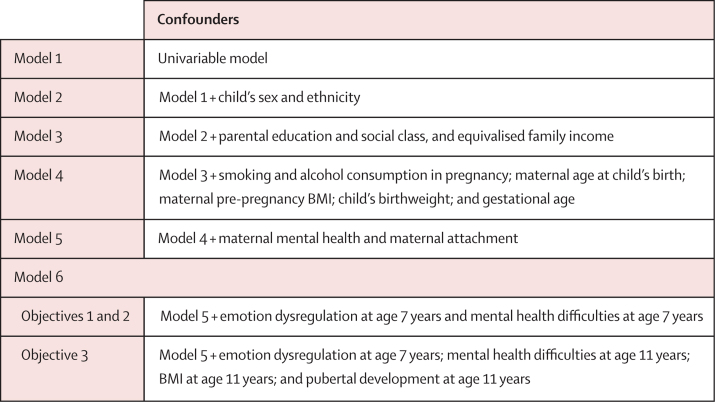
Model specification for analyses of objectives 1, 2, and 3

For objectives 1–3, we used univariable and multivariable linear regression models progressively adjusted for child-level and family-level confounders to investigate the association between exposures and outcomes (figure 1). Given previous evidence of sex-specific associations between depression and BMI, we also investigated whether these associations varied by sex. In all models we used Millennium Cohort Study population weights and accounted for sampling strata. We also ran sensitivity analyses (appendix p 8) to explore the effect on the results of particular causal assumptions and confounder measures, and calculated predicted mean outcome scores across the range of exposure values.

To investigate the extent to which body dissatisfaction at age 11 years mediated the association between BMI at age 7 years and depression at age 14 years (objective 4), we estimated the natural direct effect and the natural indirect effect of BMI on depressive symptoms (appendix p 9). These estimands allow partition of the total effect of BMI at age 7 years on depression at age 14 years into pathways that include or do not include body dissatisfaction at age 11 years by comparing potential depression scores (referred to as potential outcomes) under hypothetical interventions on BMI at age 7 years and body dissatisfaction at age 11 years (appendix p 10).

We fitted all models on participants with complete exposure data, and imputed mediator, confounder, and outcome data using multiple imputation by chained equations. We imputed 50 datasets using all variables included in the models, plus a set of auxiliary variables hypothesised to be associated with both attrition and the variables to be imputed (appendix p 10). As sensitivity analyses, we repeated all analyses using complete records. In mediation models using the *gformula* command we used single imputation using 100 imputation cycles, 1000 bootstrap samples, and 20 000 Monte Carlo simulations.

### Role of the funding source

The funders had no role in the study design, data collection, data analysis, data interpretation, or writing of the report.

## Results

Of 18 552 children included in the original cohort, 13 135 (70·8%) had BMI data available at age 7 years and were included in our sample. 6624 (50**·**4%) participants were boys and 6511 (49**·**6%) were girls. 7130 (54·5%) participants had a parent who completed compulsory education and 11 096 (84·4%) were of White ethnicity (table 1).

**Table 1:** Characteristics of study sample, based on children with complete data on BMI at age 7 years (n=13 135)

		**n (%)**	**Mean (SD)**
Total		13 135	0·51 (1·13)
Child's sex			
	Male	6624 (50·4%)	0·51 (1·19)
	Female	6511 (49·6%)	0·50 (1·08)
Child's ethnicity			
	White	11 096 (84·4%)	0·53 (1·08)
	Black	411 (3·1%)	0·89 (1·29)
	South Asian	1124 (8·6%)	0·21 (1·41)
	Mixed	351 (2·7%)	0·50 (1·27)
	Other	153 (1·2%)	0·21 (1·33)
Tercile of weekly family income			
	1st (lowest)	3786 (29·1%)	0·54 (1·22)
	2nd	4358 (33·5%)	0·55 (1·12)
	3rd (highest)	4865 (37·4%)	0·45 (1·07)
Highest parental education			
	Compulsory	7130 (54·5%)	0·57 (1·19)
	Non-compulsory	5961 (45·5%)	0·43 (1·06)
Highest parental social class			
	Professional or intermediate	8193 (68·1%)	0·47 (1·08)
	Manual or routine	3837 (31·9%)	0·60 (1·17)
Maternal pre-pregnancy BMI			
	Underweight	661 (5·5%)	0·03 (1·09)
	Healthy weight	7817 (64·9%)	0·37 (1·06)
	Overweight	2507 (20·8%)	0·75 (1·15)
	Obese	1064 (8·8%)	1·13 (1·20)
Maternal age at birth, years			
	14–20	1041 (7·9%)	0·52 (1·09)
	21–30	6002 (45·8%)	0·50 (1·14)
	31–40	5792 (44·2%)	0·51 (1·14)
	≥41	272 (2·1%)	0·54 (1·15)
Birthweight, kg			
	Low (<2·5)	886 (6·8%)	0·18 (1·23)
	Normal (≥2·5)	12 217 (93·2%)	0·53 (1·12)
Gestational age			
	Preterm	941 (7·3%)	0·42 (1·22)
	At term	12 042 (92·8%)	0·51 (1·12)
Maternal smoking in pregnancy			
	Never smoked	8682 (66·2%)	0·44 (1·12)
	Smoked but stopped	1647 (12·6%)	0·57 (1·10)
	Smoked in pregnancy	2787 (21·3%)	0·66 (1·15)
Maternal alcohol consumption in pregnancy			
	Never	8778 (69·5%)	0·52 (1·17)
	Monthly or less	2724 (21·6%)	0·47 (1·05)
	Weekly	1122 (8·9%)	0·48 (1·03)
Tercile of maternal Kessler score			
	1st (lowest symptoms)	4286 (40·3%)	0·51 (1·08)
	2nd	3749 (35·2%)	0·52 (1·10)
	3rd (highest symptoms)	2607 (24·5%)	0·53 (1·16)
Tercile of maternal attachment			
	1st (lowest scores)	5144 (46·8%)	0·46 (1·09)
	2nd	3292 (30·0%)	0·53 (1·10)
	3rd (highest scores)	2549 (23·2%)	0·59 (1·17)
Tercile of child SDQ (age 7 years)			
	1st (lowest symptoms)	4425 (34·8%)	0·51 (1·07)
	2nd	4649 (36·6%)	0·47 (1·11)
	3rd (highest symptoms)	3637 (28·6%)	0·55 (1·22)
Tercile of child emotion dysregulation (age 7 years)			
	1st (lowest symptoms)	4552 (37·3%)	0·47 (1·08)
	2nd	4977 (40·9%)	0·51 (1·12)
	3rd (highest symptoms)	2654 (21·8%)	0·58 (1·20)

Mean and SD refer to standardised BMI Z scores at age 7 years. For ease of understanding of descriptive analyses, continuous scores have been split at tertiles of their distributions to derive categorical variables. The denominator varies due to missing data. SDQ=Strengths and Difficulties Questionnaire.

At age 7 years, for the various characteristics, BMI was highest among children from Black ethnic backgrounds, those with greater emotion dysregulation, and those from households with lower parental education (table 1). Children whose mothers had higher pre-pregnancy BMI and drank alcohol or smoked during pregnancy also had higher BMI at age 7 years (table 1). At age 11 years, body dissatisfaction scores were higher in girls than boys, higher in White participants than other ethnic groups, and higher in those with higher BMI and more advanced pubertal development (appendix pp 11–12). At age 14 years, mean depressive symptom score in the sample was 5·50 (SD 5·85); girls had higher mean scores (6·98 [6·57]) than boys (3·96 [4·49]).

Among participants with complete BMI data at age 7 years (n=13 135), 1986 (15·1%) did not have data on body satisfaction and 3397 (25·9%) did not have data on depressive symptoms at age 14 years. Among those with complete exposure data, boys, children from Black ethnicity, and children whose family had a low socioeconomic position had greater proportions of missing data both at age 11 and 14 years. Children with missing data at age 11 and 14 years also had higher BMI, greater internalising and externalising symptoms and emotion dysregulation at age 7 years, and mothers with greater depressive symptoms (appendix p 13).

In the univariable model for objective 1 there was evidence that higher BMI at age 7 years was associated with greater depressive symptoms at age 14 years (estimated regression coefficient per unit increase in BMI SD 0·38, 95% CI 0·25–0·50). This association remained unchanged across subsequent models, although its magnitude was attenuated by adjustment for perinatal factors (model 4 coefficient 0·30, 0·17–0·43). Additional adjustment for maternal and child mental health did not change the results (model 6 coefficient 0·30, 0·17–0·43; table 2). There was some weak evidence (p=0·023) of an interaction between BMI and sex in model 6: the association between BMI at age 7 years and depression at age 14 years was more pronounced in girls (0·45, 0·25–0·66) than in boys (0·17, 0·00–0·33).

**Table 2:** Univariable and multivariable models estimating associations between BMI, depressive symptoms, and body dissatisfaction

		**Objective 1 outcome**		**Objective 2 outcome**		**Objective 3 outcome**	
		Coefficient for 1 SD increase in standardised BMI	p value	Coefficient for 1 SD increase in standardised BMI	p value	Coefficient for a one unit increase in body dissatisfaction score	p value
Model 1		0·38 (0·25–0·50)	<0·0001	0·17 (0·14–0·19)	<0·0001	0·83 (0·74–0·92)	<0·0001
Model 2		0·39 (0·26–0·51)	<0·0001	0·17 (0·14–0·20)	<0·0001	0·73 (0·65–0·81)	<0·0001
Model 3		0·36 (0·24–0·49)	<0·0001	0·16 (0·13–0·19)	<0·0001	0·72 (0·63–0·80)	<0·0001
Model 4		0·30 (0·17–0·43)	<0·0001	0·15 (0·12–0·18)	<0·0001	0·70 (0·62–0·78)	<0·0001
Model 5		0·31 (0·18–0·44)	<0·0001	0·15 (0·12–0·19)	<0·0001	0·69 (0·61–0·77)	<0·0001
Model 6		0·30 (0·17–0·43)	<0·0001	0·15 (0·12–0·18)	<0·0001	0·60 (0·52–0·68)	<0·0001
R^2^ model 6		9·94%	NA	5·06%	NA	14·25%	NA
BMI–sex interaction							
	Coefficient in boys	0·17 (0·00–0·33)	0·023	0·10 (0·05–0·14)	0·002	0·45 (0·34–0·56)	<0·0001
	Coefficient in girls	0·45 (0·25–0·66)	..	0·22 (0·16–0·26)	..	0·74 (0·62–0·85)	..

Objective 1 shows estimates of the association between age-standardised and sex-standardised (Z scores) BMI (kg/m^2^) at age 7 years and depressive symptoms at age 14 years. Objective 2 shows estimates of the association between age-standardised and sex-standardised (Z scores) BMI (kg/m^2^) at age 7 years and body dissatisfaction at age 11 years (scale range 0–6). Objective 3 shows estimates of the association between body dissatisfaction (scale range 0–6) at age 11 years and depressive symptoms at age 14 years. Model 1 is the univariable model. Model 2 is model 1 plus child's sex and ethnicity. Model 3 is model 2 plus highest parental education and social class, and equivalised family income. Model 4 is model 3 plus smoking and alcohol consumption in pregnancy, maternal age at child's birth, maternal pre-pregnancy BMI, child's birthweight, and gestational age. Model 5 is model 4 plus maternal mental health and maternal attachment. Model 6 is model 5 plus emotional dysregulation at age 7 years and mental health difficulties as measured with the Strengths and Difficulties Questionnaire at age 7 years. Samples for all objectives are based on participants with complete data on BMI at age 7 years and imputed confounders and outcomes (n=13 135). NA=not applicable.

In the univariable model for objective 2 there was evidence that higher BMI at age 7 years was associated with greater body dissatisfaction at age 11 years (estimated coefficient per unit increase in BMI SD 0·17, 95% CI 0·14–0·19). The estimated association remained unchanged after progressive confounder adjustment (model 6 coefficient 0·15, 0·12–0·18). There was strong evidence of an interaction between BMI and sex (p=0·002): higher BMI was associated with greater body dissatisfaction in girls (0·22, 0·16–0·26) than in boys (0·10, 0·05–0·14, table 2).

In the univariable model for objective 3 there was evidence that higher body dissatisfaction at age 11 years was associated with greater depressive symptoms at age 14 years (estimated coefficient per unit increase in dissatisfaction score 0·83, 95% CI 0·74–0·92). The magnitude of the association was attenuated after adjusting for child sociodemographic characteristics in model 2 (coefficient 0·73, 0·65–0·81), but did not further change in models 3 to 5. Adjusting for child mental health difficulties, pubertal status, and BMI at age 11 years in model 6 further reduced the magnitude of the association (0·60, 0·52–0·68), for which there was nonetheless still strong evidence (table 2). Here too we found strong evidence of an interaction between body dissatisfaction at age 11 years and sex (p<0·0001). The magnitude of the association between body dissatisfaction at age 11 years and depressive symptoms at age 14 years was larger in girls (0·74, 0·62–0·85) than boys (0·45, 0·34–0·56).

We have presented predicted outcome scores for objectives 1–3 (appendix p 14) and all sensitivity analyses (appendix pp 15–20). Results of sensitivity analyses were consistent with those reported in the main analyses.

**Figure 2:**
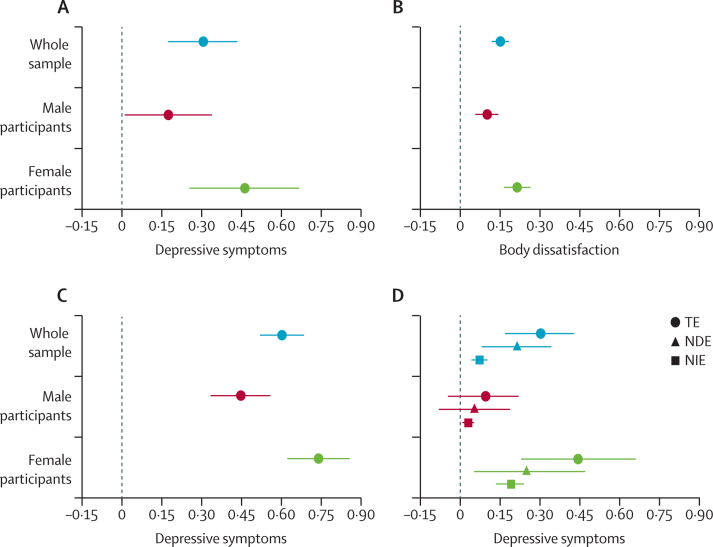
Summary of results across the four study objectives Estimates of objectives 1–3 derived from analyses in multivariable model 6. Estimates of objective 4 are derived from the fully adjusted mediation model. The x-axis represents the increase in the body dissatisfaction and depressive symptom scales, for one unit increase in the exposure (ie, 1 BMI SD or 1 point increase in body dissatisfaction scale). Body dissatisfaction is measured with a scale of 0–6 and depressive symptoms with a scale of 0–26. Objective 1: association between BMI at age 7 years and depressive symptoms at age 14 years (A); objective 2: association between BMI at age 7 years and body dissatisfaction at age 11 years (B); objective 3: association between body dissatisfaction at age 11 years and depressive symptoms at age 14 years (C); and objective 4: extent to which body dissatisfaction at age 11 years mediates the association between BMI at age 7 years and depressive symptoms at age 14 years (D). TE=total effect. NDE=natural direct effect. NIE=natural indirect effect.

In mediation analyses for objective 4, the estimated total causal effect of BMI at age 7 years on depressive symptoms at age 14 years (obtained by g-computation; coefficient per 1 SD increase 0·30, 95% CI 0·17–0·42) was consistent with those found in the initial analyses when adjusted for the same confounders. The estimated natural indirect effect was 0·08 (0·05–0·11), meaning that the proportion of the association between BMI and depression mediated by body dissatisfaction was 26% (1–52%; p=0·045; table 3, figure 2).

**Table 3:** Mediation analyses in the full sample and in the sex-stratified sample

	**Sample and estimate (95% CI)**	**p value**
**Full sample (imputed sample n=13 135)**		
Total effect	0·30 (0·17 to 0·42)	<0·0001
Natural direct effect	0·21 (0·09 to 0·34)	0·001
Natural indirect effect	0·08 (0·05 to 0·11)	<0·0001
Proportion mediated	0·26 (0·01 to 0·52)	0·045
**Girls (imputed sample n=6502)**		
Total effect	0·44 (0·23 to 0·65)	<0·0001
Natural direct effect	0·25 (0·05 to 0·46)	0·016
Natural indirect effect	0·19 (0·14 to 0·24)	<0·0001
Proportion mediated	0·43 (0·06 to 0·73)	0·005
**Boys (imputed sample n=6618)**		
Total effect	0·10 (−0·03 to 0·22)	0·136
Natural direct effect	0·06 (−0·07 to 0·19)	0·361
Natural indirect effect	0·04 (0·02 to 0·06)	0·001
Proportion mediated	0·39 (−2·98 to 3·75)	0·822

Sample based on participants with complete data on the exposure and imputed confounder, mediator, and outcome data.

Since there were sex differences in the associations examined in the previous models, we also ran sex-stratified mediation analyses. In girls, the estimated total effect of BMI on depressive symptoms was 0·44 (95% CI 0·23 to 0·65) and the natural indirect effect was 0·19 (0·14 to 0·24), representing 43% (6–73%; p=0·005) of the proportion of the main association. In boys, similarly to the earlier analyses, there was weaker evidence of an association between greater BMI and depressive symptoms (estimated total causal effect 0·09, –0·03 to 0·22), about half of which seemed to be mediated via body dissatisfaction (estimated indirect effect 0·04, 0·02 to 0·06; table 3, figure 2).

The results of the mediation analyses in the complete records sample for objective 4 were comparable to those obtained after imputation, although CIs were wider (appendix p 21).

## Discussion

We aimed to investigate the longitudinal association between childhood BMI and adolescent body dissatisfaction and depressive symptoms, and between body dissatisfaction and depressive symptoms. We also investigated the extent to which body dissatisfaction mediated the association between childhood BMI and adolescent depressive symptoms. Building on a small body of longitudinal research,^4^ in this large UK birth cohort, we found strong evidence that children with higher BMI at age 7 years had greater body dissatisfaction at age 11 years and higher depressive symptoms at age 14 years, and that those who were more dissatisfied with their appearance at age 11 years had greater depressive symptoms 3 years later.^12^, ^13^, ^14^, ^15^ For all of these associations, we observed a magnitude about twice as large in girls than in boys. In mediation analyses, body dissatisfaction explained 43% of the association between BMI and depressive symptoms in girls. In boys, estimates were comparable, but evidence of association was less clear.

These findings are in line with those of some previous studies,^17^, ^19^ but not others.^18^ The smaller effects observed in boys could be explained by different patterns of body composition in boys and girls and sex-specific societal beauty ideals. For instance, societal beauty ideals place greater value in thinness for women and on muscular bodies for men.^26^ Although BMI cannot differentiate between lean and fat mass,^27^ there is evidence that trajectories of lean and fat mass remain stable over childhood and adolescence.^28^ Therefore, if higher BMI partly captures higher lean mass, and higher lean mass represents a body type that society favours, this could have diluted the magnitude of the association between BMI and both body dissatisfaction and depressive symptoms in boys. Later onset of depression in boys could have also prevented us from observing stronger associations in analyses pertaining to objectives 1 to 3 and affected the precision of the estimates in mediation models of objective 4. To investigate these hypotheses, future studies should use various body composition measures and longer follow-up periods.

Our findings should be interpreted in light of some limitations. We measured body dissatisfaction with a single item question as opposed to a validated weight and shape concerns scale.

When exploring the association between body dissatisfaction and depressive symptoms (objective 3), we adjusted for pre-existing mental health problems at age 11 years using parent reports of internalising and externalising symptoms. At this age, parent and child reports of mental health problems might be discordant,^29^ resulting in measurement error. We conducted sensitivity analyses adjusting for a measure of self-reported mental health difficulties tapping into symptoms of depression, anxiety, and irritability. Although the magnitude of the association was slightly reduced, there was still evidence of an association, which reassured us of the robustness of our main findings. It is possible that body dissatisfaction might explain a greater proportion of the association between BMI and depressive symptoms at extreme levels of BMI (ie, very low or very high). We did not test this hypothesis as our sample would have been underpowered to run stratified mediation analyses by BMI. This hypothesis should be tested in larger samples.

Causal interpretation of our results relies on strong assumptions, including that of no residual confounding. We adjusted our model for numerous child-based and family-based confounders; however, we were not able to control for putative confounders such as parental body dissatisfaction and eating behaviours. We also did not have data on disordered eating behaviours such as binge eating. In the absence of a genetically informed design, we could not exclude genetic confounding: the effect of shared genetic heritability on these associations should be investigated. The interpretation of our estimated natural effects as causal also relies on the consistency assumption that is unlikely to be met with exposures such as BMI, which are not well defined, in the sense that changes obtained by different interventions would have the same causal effect on the outcome.^30^

Our findings suggest that greater body dissatisfaction in late childhood is an important risk factor for adolescent depression, regardless of the child's BMI. This finding is concerning. Although we observed small effect sizes, they probably translate into large population-level effects.^31^ Levels of body dissatisfaction had already increased among young people before the COVID-19 pandemic^24^ and might have further increased, given the stark rise in eating disorder presentations observed across several geographical settings since the pandemic's onset.^32^ Assuming that these associations are causal, interventions lowering body dissatisfaction at age 11 years could reduce depressive symptoms at age 14 years regardless of a child's BMI. Implementation of evidence-based body image interventions and identification of drivers of weight stigma internalisation should therefore be a key public health priority.

Interventions addressing body dissatisfaction in early adolescence exist. With some variations, they include a combination of psychological interventions and media literacy training to address putative risk factors for body dissatisfaction, such as poor self-esteem, social comparisons, negative body talk, and social media influences.^33^ These interventions show encouraging results in improving body esteem,^33^ but large trials with longer follow-ups have shown that results might not be sustained over time.^34^ Delivering these programmes alongside public health interventions targeting young people's constant exposure to a range of environmental risk factors for body dissatisfaction might help sustain the initial positive effects observed. For instance, as early as in primary school, UK children are taught about the importance of calories and exercise to maintain a healthy weight and prevent obesity, echoing population-level strategies, such as the recent inclusion of calories on restaurant and café menus. These messages, also pervasive in health-care settings, public health campaigns, and social media, might be fostering feelings of guilt, shame, and fear while placing responsibility on children for managing their weight. Further research is needed to understand how children internalise public health messaging around weight management and any association with body dissatisfaction so that existing interventions can be modified, or new ones can be developed.

Our mediation analysis findings also invite consideration around policies that target children at higher weights. For instance, in England, children are weighed in primary school both in reception year (age 4–5 years) and in year 6 (age 10–11 years) as part of the Child National Measurement Programme. Parents of children whose BMI falls in the overweight or obese categories are sent a letter advising weight loss in the child. Little support is provided to families about how and whether to discuss issues of weight beyond some general guidelines, the uptake of which is unknown. Beyond a small observational study^35^ and a randomised study investigating the uptake of child weight management, the effect of this programme on children's weight and mental health is poorly understood.^36^ There is some evidence that involving parents in universal childhood interventions might be effective in preventing excessive weight gain.^37^ However, little is known about the long-term effects of these interventions on body dissatisfaction and poor mental health outcomes, which have been shown to be cross-sectionally associated with parental restrictive dietary practices,^38^ calling for more research in this area.

Although a large proportion of the association between BMI and depressive symptoms was explained by body dissatisfaction in girls, a larger proportion of the association remained unexplained. Inflammatory pathways, shown to be associated with depressive symptoms in adolescence,^39^ could explain some of this association. However, other environmental pathways, such as bullying or weight stigma—regardless of whether these lead to weight stigma internalisation—could explain this association, perhaps via increased stress or lower self-esteem. A better understanding of these pathways could help public health efforts to reduce these effects.

Since it is a modifiable risk factor, public health strategies have focused on reducing children's BMI. Nevertheless, there is little evidence that existing interventions aimed at children and adolescents, including those used in UK schools, are effective.^40^ On the contrary, by framing weight as an individual's responsibility, these interventions might increase weight stigma internalisation and body dissatisfaction, which we found were longitudinally associated with greater depressive symptoms in adolescence, especially in girls. Our findings support increasingly frequent calls to reduce weight stigmatising messages in public health policies, health-care settings, and media to prevent negative adolescent mental health outcomes.

## Data sharing

Millennium Cohort Study data are freely available and can be downloaded on the UK Data Service website.

For the **Open Science Framework website** see https://osf.io/wsk58

**This online publication has been corrected. The corrected version first appeared at thelancet.com/psychiatry on January 18, 2024**

## Declaration of interests

We declare no competing interests.
