# Correction to *Lancet Psychiatry* 2024; 11: 47–55

**DOI:** 10.1016/S2215-0366(24)00005-1

**Published:** 2024-01-18

*Blundell E, De Stavola BL, Kellock MD et al. Longitudinal pathways between childhood BMI, body dissatisfaction, and adolescent depression: an observational study using the UK Millennium Cohort Study.* Lancet Psychiatry *2024; **11:** 47–55*—The title should have read “Millennium”. This correction has been made to the online version as of Jan 18, 2024.

