# Supplementary material for: Longitudinal pathways between childhood BMI, body dissatisfaction, and adolescent depression: an observational study using the UK Millennium Cohort Study
Source: Lancet Psychiatry. 2024 Jan;11(1):47–55. doi: 10.1016/S2215-0366(23)00365-6 (PMC11139652; doi:10.1016/S2215-0366(23)00365-6)
Supplement: Supplementary appendix [file mmc1.pdf]

THE LANCET
Psychiatry

Supplementary appendix

This appendix formed part of the original submission and has been peer reviewed. We post it as supplied by the authors.

Supplement to: Blundell E, De Stavola BL, Davies Kellock M, et al. Longitudinal pathways between childhood BMI, body dissatisfaction, and adolescent depression: an observational study using the UK Millenium Cohort Study. *Lancet Psychiatry* 2023; **10:** 47–55.

Longitudinal pathways between childhood body mass index, body dissatisfaction, and adolescent depression.

Emma Blundell[1], Bianca L. De Stavola[2], Madelaine Davies Kellock[3], Yvonne Kelly[3], Gemma Lewis[4], Anne McMunn[3], Dasha Nicholls[5], Praveetha Patalay[6], Francesca Solmi[4]

Authors' affiliations

[1] *UCL Clinical, Educational and Health Psychology*

[2] *UCL Institute of Child Health, London, UK*

[3] *UCL Institute of Epidemiology & Health, London, UK*

[4] *UCL Division of Psychiatry, London, UK.*

[5] *Division of Psychiatry, Imperial College London, London, UK*

[6] *Centre for Longitudinal Studies and MRC Unit for Lifelong Health & Ageing, UCL, London, UK*

Corresponding author

Francesca Solmi, PhD

UCL Division of Psychiatry,

Maple House, 6th Floor, wing A

149 Tottenham Court Road, W1T 7NF

London, UK.

Email: Francesca.solmi@ucl.ac.uk

Phone: + +442076799643

Supplemental material

Table of Contents

Millennium Cohort Study 4

Supplemental figure 1: distribution of Mood and Feelings Questionnaire scores in the analytical sample 4

Supplemental Figure 2: Simplified Direct Acyclic Graph 5

Confounders 6

Changes from OSF protocol 7

Sensitivity analyses 8

Estimands of Causal Mediation Analyses 9

Causal mediation analyses assumptions 10

Multiple imputation 10

Supplemental Table 1: Distribution of body dissatisfaction scores by child characteristics. Sample based on participants with available BMI at 7 and body dissatisfaction data at age 11 (n = 11,149) 11

Supplemental Table 2: Characteristics of participants with missing data on body dissatisfaction at age 11 years, and depressive symptoms at age 14 years among compared to those with available data on these outcomes among those with complete data on BMI at age 7 years (n=13,135). 13

Supplemental Table 3: predicted depressive symptoms (age 14) and body dissatisfaction (age 11) scores across BMI standardised scores (age 7). Estimates derived from adjusted model 6 presented in Table 3. 14

Supplemental Table 4: predicted depressive symptoms scores (age 14) across body dissatisfaction (age 11) scores. Estimates derived from adjusted model 6 presented in Table 4. 14

Sensitivity analyses results 15

Supplemental Table 5: Univariable and multivariable linear regression models testing the association between BMI at age 7 years and: 1) depression at age 14 years, and 2) body dissatisfaction at age 11 years. Sample based on participants with complete records on all variables included in each of the models among those with complete BMI data at age 7 years. Analyses use MCS sample weights, including non-response weights at age 14 (depression) and 11 (body dissatisfaction). 16

Supplemental Table 6: Univariable and multivariable linear regression models testing the association between body dissatisfaction at age 11 years and depression at age 14 years. Sample based on participants with complete cases on all variables included in the model. Analyses use MCS sample weights, including non-response weights at age 14. 17

Supplemental Table 7: Univariable and multivariable linear regression models testing the association between BMI at age 7 years and: 1) body dissatisfaction at age 11 years, and 2) depression at age 14 years. Sample based on participants with complete data on BMI at age 7 years (n=13,135) with adjustment for Strengths and Difficulties Questionnaire and emotion regulation score at age 5 years, instead of baseline (7 years). 18

Supplemental Table 8: Univariable and multivariable linear regression models testing the association between body dissatisfaction at age 11 years, and depression at age 14 years. Sample based on participants with complete data on BMI at age 7 years (n=13,135) with adjustment for Strengths and Difficulties Questionnaire score and BMI at age 7 years, instead of baseline (11 years). 19

Supplemental Table 9: Univariable and multivariable linear regression models testing the association between body dissatisfaction at age 11 years, and depression at age 14 years. Sample based on participants with complete data on BMI at age 7 years (n=13,135). Model 6 adjusted for self-reported mental health at age 11 years, instead of mother reported SDQ score. 20
Supplemental Table 10: Mediation analyses on complete records (n=5,147) 21
Stata code for causal mediation analysis 22
References 23

Millennium Cohort Study

The Millennium Cohort Study is a longitudinal UK birth cohort study, which aimed to recruit all children born between 1 September 2000 and 31 August 2001 (England and Wales), and between 24 November 2000 and 11 January 2002 (Scotland and Northern Ireland), who were living in the UK at age 9 months and were eligible for universal child benefit.

The Millennium Cohort Study used a clustered stratified sampling design to ensure over-representation of children from ethnic minority and deprived backgrounds, traditionally harder to reach. The initial sample included 18,552 families (72% of all eligible) and 18,562 singletons, 246 sets of twins, and 10 sets of triplets. Participants have been since followed up 7 times at 9 months, and at 3, 5, 7, 11, 14, and 17 years. The sample was supplemented at the second wave with 692 families who were initially eligible for inclusion but who did not participate[1].

Supplemental Figure 1: distribution of Mood and Feelings Questionnaire scores in the analytical sample

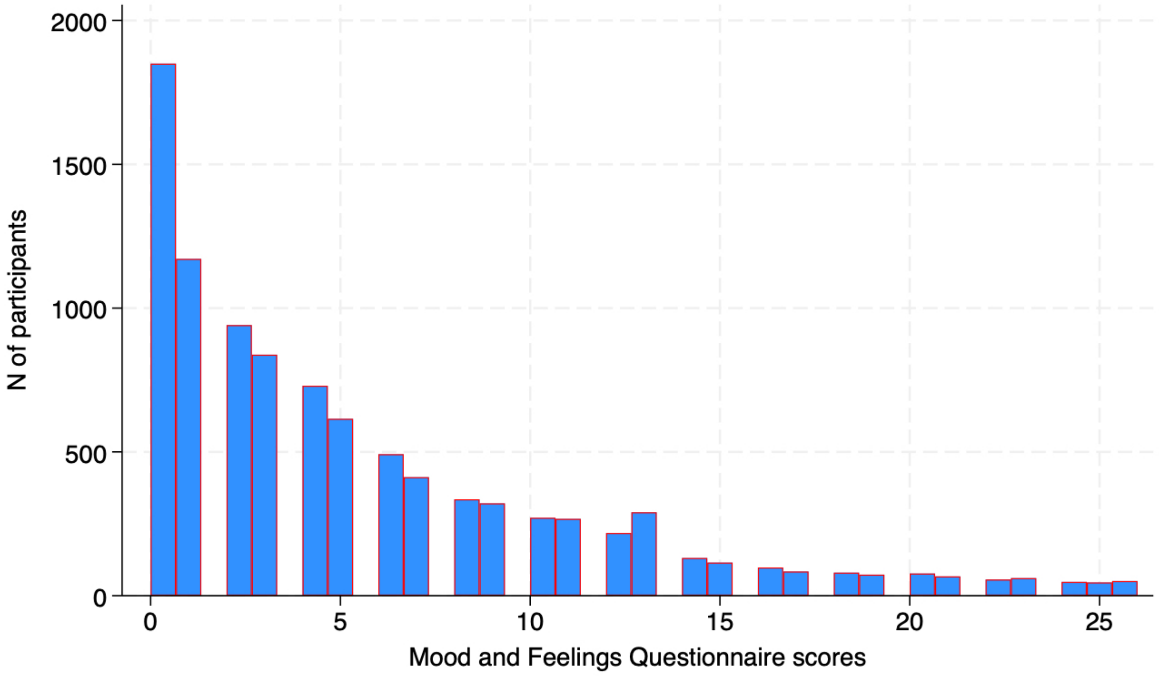

Supplemental Figure 2: Simplified Direct Acyclic Graph

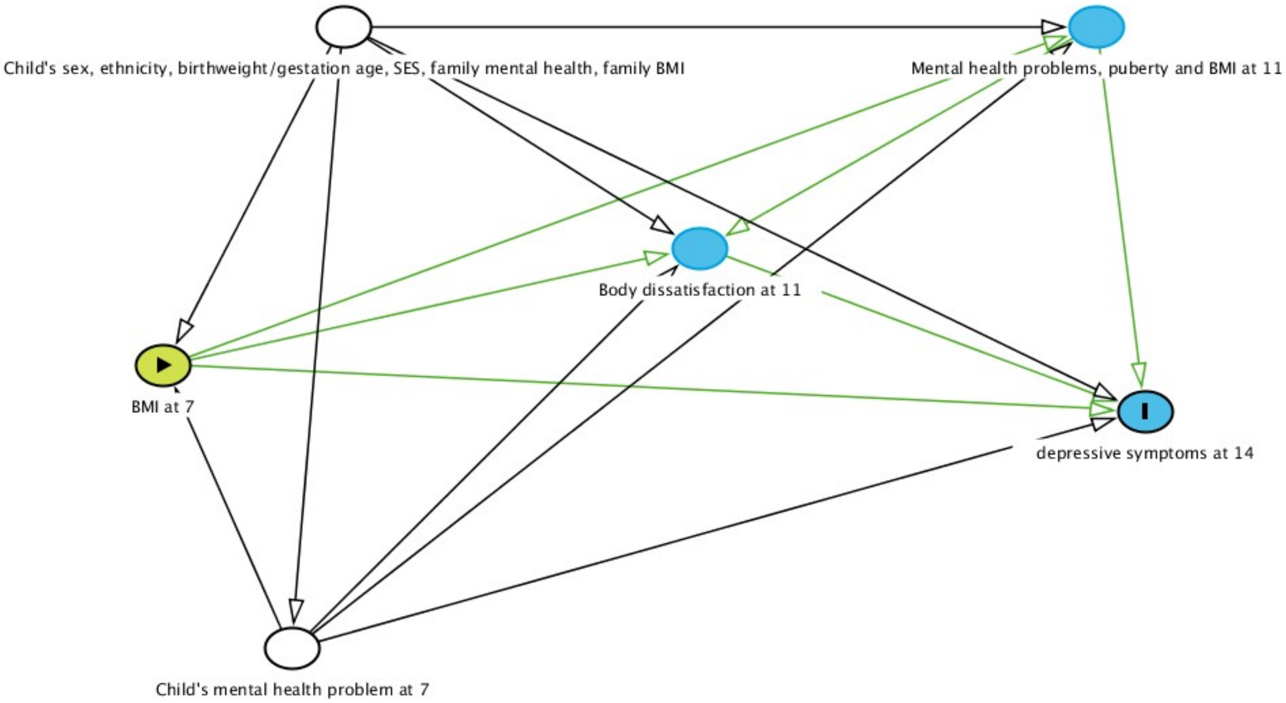

Confounders

We identified a set of confounders which were common across analyses investigating objectives 1-3.

- Model 2 adjusted for child's sex at birth (male/female) and ethnicity. The latter was coded according to Office of National Statistics categorisation of White (White British, White other), Black (Black Caribbean, Black African), Asian (Indian, Pakistani, Bangladeshi), mixed ethnicity, other; as reported by the mother when the child was 9 months.
- In Model 3 we included highest parental academic qualification (coded as " up to compulsory" vs "non-compulsory"), social class (recoded from National Statistics Socio-Economic Categories into "managerial/intermediate/self-employed" vs "low supervisory /technical /routine/semi-routine") and household OECD-equivalised weekly income. We included these variables as there is evidence that more deprived groups have higher BMI[2] and higher mental health problems including symptoms of depression[3] and eating disorders[4]. These variables were self-reported by the mothers when the child was 9 months. For the variables 'highest family academic qualification' and 'social class', in case of single parent-households or two-parent households in which either had missing data we retained the observed value.
- Model 4 further adjusted for a number of perinatal factors including smoking in pregnancy ("never smoked", "smoked but stopped in pregnancy", "smoked in pregnancy") and alcohol consumption in pregnancy ("Never drank", "drank monthly or less frequently", "drank weekly"), child's birthweight in grams and gestational age in weeks, and maternal age and pre-pregnancy BMI. These variables were included because of observed associations with offspring BMI[5,6], depression[7] (or risk factors for depression[8]), and eating disorders[9,10] or because we thought they could capture unmeasured parental characteristics (i.e. risk taking) which could reflect genetic risk and/or other environmental risk pathways even in absence of specific causal associations[11]. These measures were reported by mothers when the child was 9 months old.
- In Model 5 we adjusted for maternal depressive symptoms at child age 3 years using the Kessler-6 scale[12] and for maternal attachment at child age 9 months measured using the sum of six items from the Condon maternal attachment questionnaire[13], as these variables have evidence of associations with BMI[14], depression[15], and eating disorders[16].
- In Model 6: we adjusted for child's mother-reported emotion dysregulation at age 7 years due to known associations with higher BMI, depressive symptoms and eating

disorders.[17–19]. Mothers reported on their child's emotion regulation abilities over the previous six-months using five questions adapted from Adaptive Social Behaviour Inventory and included in the Child Social Behaviour Questionnaire.[20]

Model-specific confounders were the following:
In Model 6 we adjusted for internalising and externalising problems were measured with the Strengths and Difficulties Questionnaires a 20-item questionnaire capturing emotion and peer problems (internalising symptoms) and hyperactivity and conduct problems (externalising symptoms). In objective 1 and 2, these were measured at age 7 years, in objective 3 at age 11 years. We included mental health difficulties due to putative associations with BMI and body dissatisfaction[21,22] (see also *Sensitivity analyses*). In objective 4, we also adjusted analyses in model 6 for child BMI at age 11 (measured and age- and sex-standardised as in the main exposure at 7 years) and self-reported pubertal status at age 11 given putative associations with BMI[23], depression[24], and eating disorders[25]. Participants were asked to describe changes in skin, hair and growth spurt; girls were further asked about changes in breasts and boys about changes in voice. Possible answers were: 0='not started', 1='barely started', 2='definitely started'. From these questions we created a total score with higher scores indicating greater pubertal development.

Changes from OSF protocol

In the final manuscript we no longer included parental conflict and paternal depression as confounders, because we realised we would have had to exclude children from single-parent households or impute a value for children whose variable did not apply to, both of which could have potentially biased results. We also no longer adjusted for school readiness score after deliberations on the lack of theoretical support for the inclusion of this variable. We report no other changes from protocol.

Sensitivity analyses

We ran a number of sensitivity analyses.

First, we re-ran all main analyses pertaining to objectives 1, 2, and 3 on participants with complete data on all variables used in the analyses, in order to compare results with those from multiply imputed datasets.

Second, we re-fitted model 6 adjusting for mental health difficulties emotion regulation at age 5 years (objective 1 and 2), and BMI and mental health difficulties at age 7 years (objective 3), i.e. at the wave of data collection preceding that of the exposure measurement. In the main models we had adjusted for these confounders as measured at baseline (i.e. age 7 years for objectives 1, and 2; and age 11 years for objective 3) under the assumption that difficulties with emotion regulation[26] (Objectives 1 & 2), and mental health difficulties [17,21,22] problems can lead to higher BMI and body dissatisfaction (Objectives 1, 2, 3), particularly in light of emerging evidence that childhood BMI might not be associated with increased childhood mental health difficulties.[27] Similarly, we adjusted for BMI at 11 in Objective 3 as body dissatisfaction is more common in those with higher BMI.[28] We ran these sensitivity analyses because there is also some evidence internalising problems could be a consequence of both BMI and body dissatisfaction (which is also in line with our own hypothesis)[29,30] and there is evidence that externalising symptoms could be caused by higher BMI.[31] Similarly, it has been hypothesised that body dissatisfaction might lead to weight gain.[32] Under these competing assumptions, adjusting for baseline levels of these factors could lead to over-adjustment, as these would be on the causal pathway between exposure and outcome. Adjusting for these factors at the previous wave, reduces this possibility.[33]

Finally, we re-fitted model 6 for objective 3 adjusting for self-reported, instead of parent-reported, mental health difficulties at age 11 years as there is some evidence parents might underreport symptoms when children are adolescent[34] leading to poorer confounder adjustment. We have not included this measure in the main analyses as the Strengths and Difficulties Questionnaire is a well-validated mental health questionnaire.

Estimands of Causal Mediation Analyses

Natural direct effect (NDE) of BMI on depressive symptoms, not mediated by body dissatisfaction at 11. This is defined as: E[$Y_{a,M(a^*)}$ – $Y_{a^*,M(a^*)}$]. Where E stands for the expectation over the population of interest, *Y* represents the outcome, *A* the exposure, and *M* the mediator. Here, $Y_{a,M(a^*)}$ represents potential depressive symptoms *Y* had BMI been set to take the value a, while at the same time body dissatisfaction *M* had been set to take the natural value that would have occurred had depressive symptoms been set to take the value A=a*. $Y_{a^*,M(a^*)}$ represents potential depressive symptoms had BMI been set to take the value a* and body dissatisfaction as before been set to take the natural value that would occur had BMI been set at the value A=a* . In other words, this is the mean difference between the counterfactual outcomes if participants were exposed to the BMI value *A*=a compared to BMI value a*, while in both settings the mediator were set to take the natural value it would have when the exposure *A*=a*.

Natural indirect effect (NIE) of BMI on depressive symptoms, mediated via the effect on body dissatisfaction at 11. This is defined as E[$Y_{a,\ M(a)}$ – $Y_{a,M(a^*)}$]. Here, $Y_{a,M(a)}$ represents potential depressive symptoms *Y* had BMI been set to take the value A=a, and M had been set to take the natural value it would take if the exposure *A* =a; and $Y_{a,M(a^*)}$ represents potential depressive symptoms *Y* had BMI been set to take the value a, and *M* had been set to take the value that would have occurred had depressive symptoms been set to the value of a*. This is the mean difference between the counterfactual outcomes when the exposure is set to *A*=a in both settings but the mediator had been set to whatever value it would have taken at a reference value of the exposure A = a* .

Total effect (TE) of BMI on depressive symptoms is defined as the causal contrast: E[$Y_{a,\ M(a)}$ – $Y_{a^*,M(a^*)}$]. Here, $Y_{a,\ M(a}$and $Y_{a^*,M(a^*)}$ are defined above. This is the mean difference between the counterfactual outcomes when the exposure is set to *A*=a and when the exposure is set to A=a* and, in each case, the mediator had also been set to whatever value it would have taken at the same exposure level (respectively a and a*).

Proportion mediated is calculated as NIE/TE and reported as a percentage.

Confidence intervals for all estimates of these quantities were obtained by bootstrap.

Causal mediation analyses assumptions

Estimands from causal mediation analyses can be identified under the technical assumptions of: (1) no interference of the potential outcome and potential mediator of one individual by the exposure of another individual, and (2) consistency for the potential outcomes and potential mediators. In the context of our analyses, the first assumption implies that the BMI of one child does not influence the body dissatisfaction or depression symptoms score of another. The second assumption implies that how changes in BMI are achieved does not change their potential impact on, respectively, body dissatisfaction and depression score (and similarly for changes in body dissatisfaction on depression). In addition, (3) the assumption of no unmeasured confounding of the exposure-mediator, exposure-outcome and mediator-outcome relationships is invoked when estimating these quantities using g-computation as implemented in the "*gformula*" command in Stata with 20,000 Monte Carlo simulations, and confidence intervals derived from 1000 bootstrap samples .

Multiple imputation

For participants with complete exposure data, we imputed missing values in mediator and outcome variables using multiple imputation with chained equations when addressing objectives 1-3. We imputed 50 datasets using linear, logistic, and multinomial logistic regression according to the nature of the variables. In the models, we included all variables included in the main models as well as a number of auxiliary variables. The latter were: BMI at age 3, 5, and 14; Strengths and Difficulties questionnaire total score at ages 3, 5, and 14, maternal self-esteem, sample weights; self-regulation measures at 3, 5, and 7; emotion regulation measures at 3 and 5; body dissatisfaction at age 14; paternal depression; and child's Bracken reading scores at age 5 years. Single imputation with the same imputation models as above was used in mediation analyses because inference was obtained by bootstrap.

Supplemental Table 1: Distribution of body dissatisfaction scores by child characteristics. Sample based on participants with available BMI at 7 and body dissatisfaction data at age 11 (n = 11,149)

	**Body dissatisfaction score age 11 years**
	Mean (SD)
**Total**	1.49 (1.62)
**Child's sex**	
Male	1.35 (1.58)
Female	1.64 (1.65)
**Child's ethnicity**	
White	1.54 (1.62)
Black	1.33 (1.77)
South Asian	1.13 (1.53)
Mixed	1.41 (1.65)
Other	1.13 (1.55)
**Thirds of weekly family income**	
1st (lowest)	1.52 (1.76)
2nd	1.54 (1.64)
3rd (highest)	1.44 (1.50)
**Highest parental education**	
Compulsory	1.55 (1.71)
Non-compulsory	1.43 (1.51)
**Highest parental social class**	
Professional / Intermediate	1.45 (1.53)
Manual / Routine	1.63 (1.78)
**Maternal pre-pregnancy BMI**	
Underweight	1.46 (1.69)
Normal weight	1.44 (1.57)
Overweight	1.55 (1.68)
Obese	1.79 (1.74)
**Maternal age at birth**	
14-20 years	1.62 (1.76)
21-30 years	1.50 (1.66)
31-40 years	1.47 (1.57)
41 years+	1.54 (1.48)
**Birth weight**	
Low (< 2.5kg)	1.52 (1.76)
Normal (≥2.5kg)	1.49 (1.61)
**Gestational age**	
Preterm	1.48 (1.64)
At term	1.50 (1.62)
**Maternal smoking in pregnancy**	
Never smoked	1.41 (1.56)
Smoked but stopped	1.65 (1.67)
Smoked in pregnancy	1.66 (1.78)
**Maternal alcohol consumption in pregnancy**	
Never	1.46 (1.63)
Monthly or less	1.58 (1.60)
Weekly	1.56 (1.60)

Supplemental Table 1 (continued)

	**Body dissatisfaction score age 11 years**
	Mean (SD)
**Thirds of maternal Kessler score**	
1st (lowest symptoms)	1.39 (1.56)
2nd	1.56 (1.60)
3rd (highest symptoms)	1.65 (1.71)
**Thirds of maternal attachment**	
1st (lowest scores)	1.54 (1.60)
2nd	1.55 (1.65)
3rd (highest scores)	1.38 (1.58)
**Thirds of child SDQ (age 7)**	
1st (lowest symptoms)	1.34 (1.47)
2nd	1.48 (1.60)
3rd (highest symptoms)	1.73 (1.80)
**Thirds of child emotion dysregulation (age 7)**	
1st (lowest symptoms)	1,35 (1.48)
2nd	1.53 (1.63)
3rd (highest symptoms)	1.75 (1.81)

Supplemental Table 2: Characteristics of participants with missing data on body dissatisfaction at age 11 years, and depressive symptoms at age 14 years among compared to those with available data on these outcomes among those with complete data on BMI at age 7 years (n=13,135).

	**Missing body dissatisfaction data at age 11 years**		**Missing depression data at age 14 years**	
	No n= 11,149 (84.9%)	*Yes n= 1,986 (15.1%)*	*No n= 9,738 (74.1%)*	*Yes n= 3,397 (25.9%)*
	n (%)	***n (%)***	***n (%)***	***n (%)***
**Child's sex**				
Male	5,507 (83.2)	1,117(16.9)	4,771 (72.0)	1,853 (28.0)
Female	5,642 (86.7)	869 (13.4)	4,967 (76.3)	1,544 (23.7)
**Child's ethnicity**				
White	9,468 (85.3)	1,628 (14.7)	8,177 (73.7)	2,919 (26.3)
Black	303 (73.7)	108 (26.3)	280 (68.1)	131 (31.9)
South Asian	958 (85.2)	166 (14.8)	904 (80.4)	220 (19.6)
Mixed	293 (83.5)	58 (16.5)	252 (72.0)	99 (28.0)
Other	127 (83.0)	26 (17.0)	125 (81.7)	28 (18.3)
**Parent highest education**				
Compulsory	5,809 (81.5)	1,321 (18.6)	4,876 (68.3)	2,254 (31.7)
Non-compulsory	5,309 (89.1)	652 (10.9)	4,837 (81.1)	1,124 (18.9)
**Parent highest social class**				
Professional / Intermediate	7,201 (88.9)	992 (12.1)	6,462 (78.9)	1,731 (21.1)
Manual / Routine	3,090 (80.5)	747 (19.5)	2,540 (66.2)	1,297 (33.8)
**Maternal smoking in pregnancy**				
Never smoked	7,544 (86.9)	1,138 (13.1)	6,757 (77.8)	1,925 (22.2)
Smoked but stopped in pregnancy	1,361 (82.6)	286 (17.4)	1,149 (69.8)	498 (30.2)
Smoked in pregnancy	2,231 (80.0)	556 (20.0)	1,821 (65.4)	966 (34.6)
**Maternal drinking in pregnancy**				
Never	7,381 (84.1)	1,397 (15.9)	6,4013(73.1)	2,365 (26.9)
Monthly or less	2,345 (86.1)	379 (13.9)	2,059 (75.6)	665 (24.4)
Weekly	991 (88.3)	131 (11.7)	872 (77.7)	250 (22.3)
	**Mean (SD)**	**Mean (SD)**	**Mean (SD)**	**Mean (SD)**
**BMI at age 7**	0.48 (1.12)	0.63 (1.17)	0.47 (1.11)	0.61 (1.19)
**BMI at age 11**	0.57 (1.21)	0.63 (1.27)	0.55 (1.20)	0.67 (1.27)
**Family income**	315.18 (202.78)	257.11 (183.83)	322.11 (205.51)	261.40 (180.55)
**Maternal pre-pregnancy BMI**	23.77 (4.43)	23.53 (4.45)	23.76 (4.41)	23.66 (4.51)
**Maternal age at birth (years)**	29.81 (5.75)	28.21 (6.21)	30.02 (5.70)	28.30 (6.10)
**Child's birthweight (kg)**	3.36 (0.58)	3.34 (0.59)	3.37 (0.58)	3.34 (0.59)
**Gestational age (weeks)**	39.43 (1.93)	39.36 (2.04)	39.45 (1.92)	39.34 (2.03)
**Maternal depression (Kessler scale)**	3.16 (3.62)	3.40 (3.92)	3.09 (3.52)	3.52 (4.07)
**Maternal attachment**	18.50 (2.50)	18.79 (2.56)	18.46 (2.51)	18.78 (2.48)
**Child SDQ score at age 7**	7.26 (5.31)	8.46 (5.88)	7.09 (5.17)	8.44 (5.96)
**Child emotion dysregulation at age 7**	3.51 (2.35)	3.99 (2.42)	3.45 (2.34)	3.96 (2.41)

Supplemental Table 3: predicted depressive symptoms (age 14) and body dissatisfaction (age 11) scores across BMI standardised scores (age 7). Estimates derived from adjusted model 6 presented in Table 3.

Standardised BMI score	Mean depressive symptoms score (95% CI)	Mean body dissatisfaction scores (95% CI)
-4	4.28 (3.69 to 4.87)	0.88 (0.72 to 1.03)
-3	4.57 (4.11 to 5.04)	1.03 (0.91 to 1.15)
-2	4.87 (4.53 to 5.22)	1.17 (1.08 to 1.26)
-1	5.17 (4.94 to 5.40)	1.32 (1.26 to 1.38)
0	5.47 (5.32 to 5.61)	1.47 (1.43 to 1.51)
1	5.76 (5.61 to 5.92)	1.62 (1.58 to 1.66)
2	6.06 (5.81 to 6.31)	1.77 (1.71 to 1.83)
3	6.36 (6.01 to 6.72)	1.92 (1.83 to 2.01)
4	6.66 (6.18 to 7.13)	2.07 (1.95 to 2.19)

Supplemental Table 4: predicted depressive symptoms scores (age 14) across body dissatisfaction (age 11) scores. Estimates derived from adjusted model 6 presented in Table 4.

Body dissatisfaction score	Mean depressive symptoms score (95% CI)
0	4.68 (4.50 to 4.85)
1	5.28 (5.14 to 5.42)
2	5.88 (5.74 to 6.02)
3	6.48 (6.30 to 6.67)
4	7.08 (6.83 to 7.34)
5	7.68 (7.36 to 8.01)
6	8.28 (7.88 to 8.68)

Sensitivity analyses results

Complete case analyses: In complete case analyses of objectives 1, 2, and 3, results remained comparable to those obtained in analyses using imputed datasets, although at times confidence intervals were broader, likely due to the smaller sample sizes. Results of stratified analyses were also consistent with those of the main analyses (Supplemental Table 2 and Supplemental Table 3).

Model 6 adjustment for confounders at the previous wave: Adjusting for mental health difficulties and emotion dysregulation at age 5 instead of 7 years in analyses of objective 1 and 2, and mental health difficulties and BMI at age 7 instead of 11 in analyses of objective 3 also did not alter results (Supplemental Table 4 and Supplemental Table 5).

Using self-reported mental health at age 11 years. In objective 3 analyses (Supplemental Table 6), when we adjusted model 6 for self-reported (instead of maternal reported) mental health difficulties in there was a reduction in the magnitude of the association between body dissatisfaction at 11 years and depressive symptoms at 14 years (coefficient 0.47, 95%CI: 0.39, 0.56) although there was still strong evidence of an association. This suggests that using parental-report of mental health difficulties at age 11 years might lead to residual confounding, if their reporting is affected by error, e.g. by underestimating adolescent mental health difficulties.

Supplemental Table 5: Univariable and multivariable linear regression models testing the association between BMI at age 7 years and: 1) depression at age 14 years, and 2) body dissatisfaction at age 11 years. Sample based on participants with complete records on all variables included in each of the models among those with complete BMI data at age 7 years. Analyses use MCS sample weights, including non-response weights at age 14 (depression) and 11 (body dissatisfaction).

	**Objective 1 outcome: Depression 14 years,**	**Objective 2 outcome Body dissatisfaction 11 years**
	**Coefficient for one standard deviation increase in standardised BMI (95% CI) p-value N=5,790**	**Coefficient for one standard deviation increase in standardised BMI (95% CI) p-value N=5,516**
**Model 1**<br>Univariable model	0.38 (0.23 to 0.54)<br>p<0.0001	0.15 (0.12 to 0.19)<br>p<0.0001
**Model 2**<br>Model 1 + child sex and ethnicity	0.41 (0.27 to 0.56)<br>p<0.0001	0.15 (0.12 to 0.19)<br>p<0.0001
**Model 3:**<br>Model 2 + highest education; social class; weekly family income	0.39 (0.24 to 0.53)<br>p<0.0001	0.15 (0.11 to 0.18)<br>p<0.0001
**Model 4**<br>Model 3 + smoking and alcohol consumption in pregnancy; maternal age at child's birth; maternal pre-pregnancy BMI; child's birthweight; gestational age	0.29 (0.14 to 0.44)<br><0.0001	0.13 (0.09 to 0.17)<br>p<0.0001
**Model 5**<br>Model 4 + maternal depression; maternal attachment	0.30 (0.15 to 0.45)<br>p<0.0001	0.13 (0.09 to 0.17)<br>p<0.0001
**Model 6**<br>Model 5 + emotion dysregulation at age7, SDQ at age 7	0.30 (0.15 to 0.45)<br>p<0.001	0.13 (0.09 to 0.17)<br>p<0.0001
	**R^2 (model 6)**	**R^2 (model 6)**
	9.55%	4.45%
	**p-value for interaction**	**p-value for interaction**
**BMI*sex interaction added to Model 6**	**p=0.016**	**P=0.001**
**Coefficient in** boys	0.13 (-0.01 to 0.27)	0.06 (0.01 to 0.11)
**Coefficient in** girls	0.49 (0.23 to 0.76)	0.22 (0.16 to 0.27)

Supplemental Table 6: Univariable and multivariable linear regression models testing the association between body dissatisfaction at age 11 years and depression at age 14 years. Sample based on participants with complete cases on all variables included in the model. Analyses use MCS sample weights, including non-response weights at age 14.

	**Objective 3 outcome: Depression, 14 years**
	**Coefficient for a one-point increase in body dissatisfaction score (95% CI) p-value N=5,202**
**Model 1**<br>Univariable model	0.83 (0.572to 0.93) p<0.0001
**Model 2**<br>Model 1 + child sex and ethnicity	0.75 (0.65 to 0.85) p<0.0001
**Model 3:**<br>Model 2 + highest education; social class; weekly family income	0.74 (0.47 to 0.80) p<0.0001
**Model 4**<br>Model 3 + smoking and alcohol consumption in pregnancy; maternal age at child's birth; maternal pre-pregnancy BMI; child's birthweight; gestational age	0.72 (0.61 to 0.82) p<0.0001
**Model 5**<br>Model 4 + parental mental health; marital conflict; maternal attachment	0.71 (0.61 to 0.81) p<0.0001
**Model 6**<br>Model 5 + emotion dysregulation at age7; SDQ at age 11; BMI at age 11; pubertal development at age 11	0.68 (0.58 to 0.79) p<0.0001
	**R^2 (model 6)**
	12.25%
	**p-value for interaction**
**Body dissatisfaction*sex interaction added to Model 6**	**P<0.0001**
**Coefficient in** boys	0.43 (0.30 to 0.56)
**Coefficient in** girls	0.92 (0.76 to 1.07)

Supplemental Table 7: Univariable and multivariable linear regression models testing the association between BMI at age 7 years and: 1) body dissatisfaction at age 11 years, and 2) depression at age 14 years. Sample based on participants with complete data on BMI at age 7 years (n=13,135) with adjustment for Strengths and Difficulties Questionnaire and emotion regulation score at age 5 years, instead of baseline (7 years).

	**Objective 1 outcome: Depressive symptoms 14 years**	**Objective 2 outcome: Body dissatisfaction, 11 years**
	**Coefficient for one standard deviation increase in standardised BMI (95% CI) p-value**	**Coefficient for one standard deviation increase in standardised BMI (95% CI) p-value**
**Model 1**<br>Univariable model	0.38 (0.25 to 0.50)<br>p<0.0001	0.17 (0.14 to 0.19)<br>p<0.0001
**Model 2**<br>Model 1 + child's sex and ethnicity	0.39 (0.26 to 0.51)<br>p<0.0001	0.17 (0.14 to 0.20)<br>p<0.0001
**Model 3:**<br>Model 2 + highest education; social class; weekly family income	0.36 (0.24 to 0.49)<br>p<0.0001	0.16 (0.13 to 0.19)<br>p<0.0001
**Model 4**<br>Model 3 + smoking and alcohol consumption in pregnancy; maternal age at child's birth; maternal pre-pregnancy BMI; child's birthweight; gestational age	0.30 (0.17 to 0.43)<br>p<0.0001	0.15 (0.12 to 0.18)<br>p<0.0001
**Model 5**<br>Model 4 + maternal mental health; maternal attachment	0.31 (0.18 to 0.44)<br>p<0.0001	0.15 (0.12 to 0.19)<br>p<0.0001
**Model 6**<br>Model 5 + SDQ at age 5; emotional dysregulation at age 5	0.30 (0.17 to 0.43)<br>P<0.0001	0.15 (0.11 to 0.18)<br>P<0.0001
	**R^2 (model 6)**	**R^2 (model 6)**
	9.67%	4.40%
	**p-value for interaction**	**p-value for interaction**
**BMI*sex interaction added to Model 6**	**p=0.017**	**P=0.001**
**Coefficient in** boys	0.16 (0.01 to 0.33)	0.10 (0.05 to 0.14)
**Coefficient in** girls	0.46 (0.25 to 0.66)	0.21 (0.16 to 0.26)

Supplemental Table 8: Univariable and multivariable linear regression models testing the association between body dissatisfaction at age 11 years, and depression at age 14 years. Sample based on participants with complete data on BMI at age 7 years (n=13,135) with adjustment for Strengths and Difficulties Questionnaire score and BMI at age 7 years, instead of baseline (11 years).

	**Objective 3 outcome: Depression, 14 years**
	**Coefficient for a one-point increase in body dissatisfaction score (95% CI) p-value**
**Model 1**<br>Univariable model	0.83 (0.74 to 0.92)<br>p<0.0001
**Model 2**<br>Model 1 + child sex and ethnicity	0.73 (0.65 to 0.81)<br>p<0.0001
**Model 3:**<br>Model 2 + highest education; social class; weekly family income	0.72 (0.63 to 0.80)<br>p<0.0001
**Model 4**<br>Model 3 + smoking and alcohol consumption in pregnancy; maternal age at child's birth; maternal pre-pregnancy BMI; child's birthweight; gestational age	0.70 (0.62 to 0.78)<br>p<0.0001
**Model 5**<br>Model 4 + maternal depression, maternal attachment	0.69 (0.61 to 0.77)<br>p<0.0001
**Model 6**<br>Model 5 + emotion dysregulation at age7, SDQ at age 7; BMI at age 7; pubertal development at age 11	0.65 (0.57 to 0.73)<br>P<0.0001
	**R^2 (model 6)**
	12.95%
	**p-value for interaction**
**Body dissatisfaction*sex interaction added to Model 6**	**p<0.0001**
**Coefficient in** boys	0.47 (0.36 to 0.58)
**Coefficient in** girls	0.80 (0.69 to 0.92)

Supplemental Table 9: Univariable and multivariable linear regression models testing the association between body dissatisfaction at age 11 years, and depression at age 14 years. Sample based on participants with complete data on BMI at age 7 years (n=13,135). Model 6 adjusted for self-reported mental health at age 11 years, instead of mother reported SDQ score.

	**Objective 3 outcome: Depression, 14 years**
	**Coefficient for a one-point increase in body dissatisfaction score (95% CI) p-value**
**Model 1**<br>Univariable model	0.83 (0.74 to 0.92)<br>p<0.0001
**Model 2**<br>Model 1 + child sex and ethnicity	0.73 (0.65 to 0.81)<br>p<0.0001
**Model 3:**<br>Model 2 + highest education; social class; weekly family income	0.72 (0.63 to 0.80)<br>p<0.0001
**Model 4**<br>Model 3 + smoking and alcohol consumption in pregnancy; maternal age at child's birth; maternal pre-pregnancy BMI; child's birthweight; gestational age	0.70 (0.62 to 0.78)<br>p<0.0001
**Model 5**<br>Model 4 + parental mental health; marital conflict; maternal attachment	0.69 (0.61 to 0.77)<br>p<0.0001
**Model 6**<br>Model 5 + emotion dysregulation at age7, self-reported mental health at age 11; BMI at age 11; pubertal development at age 11	0.47 (0.39 to 0.56)<br>p<0.0001
	**R^2 (model 6)**
	15.98%
	**p-value for interaction**
**Body dissatisfaction*sex interaction added to Model 6**	**p<0.0001**
**Coefficient in** boys	0.37 (0.26 to 0.48)
**Coefficient in** girls	0.55 (0.44 to 0.68)

Supplemental Table 10: Mediation analyses on complete records (n=5,147)

**Estimand**	**Sample and Estimate (95% CI), p-value**
**Full sample**	**Complete cases, n=5,147**
Total Effect	0.27 (0.12, 0.43), p=0.025
Natural Direct Effect	0.19 (-0.05, 0.42), p=0.121
Natural Indirect Effect	0.09 (0.04, 0.13), p<0.0001
Proportion mediated	0.32 (-6.27, 6.89), p=0.925
**Girls**	**Complete cases, n=2,648**
Total Effect	0.38 (0.10, 0.65), p=0.007
Natural Direct Effect	0.20 (-0.07, 0.47), p=0.147
Natural Indirect Effect	0.17 (0.10, 0.24), p<0.0001
Proportion mediated	0.48 (-0.25, 1.18), p=0.869
**Boys**	**Complete cases, n=2,499**
Total Effect	0.17 (0.01, 0.34), p=0.047
Natural Direct Effect	0.15 (-0.02, 0.32), p=0.091
Natural Indirect Effect	0.03 (-0.01, 0.05), p=0.080
Proportion mediated	0.15 (-1.93, 2.22), p=0.891

Stata code for causal mediation analysis

<u>Outcome</u>
y= depressive symptoms (outcome)

<u>Exposure</u>
x= BMI at age 7 (exposure)

<u>Mediator</u>
m= body dissatisfaction at age 11 (mediator)

<u>Intermediate confounders</u>
I1=BMI at age 11
I2= pubertal status at age 11
I3= strengths and difficulties questionnaire score at age 11

<u>Confounders ($confs)</u>
Sex; ethnicity; maternal age; parental highest education; Parental highest social class; family weekly equivalised income; maternal pre-pregnancy BMI; Child's birthweight and gestational weeks, maternal depression; maternal smoking in pregnancy; maternal drinking in pregnancy; maternal attachment; Strengths and Difficulties questionnaire total score at age 7, emotion dysregulation at age 7

<u>Mediation analyses code in complete records:</u>
```
preserve
#delimit ;
gformula y x m I1 I2 I3 $confs,
mediation
outcome(y) exposure(x)  mediator(m) post_confs(I1 I2 I3)
base_confs($confs )
commands(y: regress, m: regress, I1: regress, I2: regress, I3: regress)
equations
                    (y : x m I3 I2 I1         $confs  ,
                     m: x    I3 I2 I1         $confs  ,
                     I3: x      I2 I1         $confs  ,
                     I2: x         I1         $confs  ,
                     I1: x                    $confs  ,
                     )
control(m:0) linexp
samples(1000) moreMC simulations(20000) replace seed(79)
;
#delimit cr
Restore
```

References

1 Connelly R, Platt L. Cohort Profile: UK Millennium Cohort Study (MCS). *International Journal of Epidemiology* 2014; **43**: 1719–25.

2 Devaux M, Sassi F. Social inequalities in obesity and overweight in 11 OECD countries. *European Journal of Public Health* 2013; **23**: 464–9.

3 Hazell M, Thornton E, Haghparast-Bidgoli H, Patalay P. Socio-economic inequalities in adolescent mental health in the UK: Multiple socio-economic indicators and reporter effects. *SSM - Mental Health* 2022; **2**: 100176.

4 Micali N, De Stavola B, Ploubidis G, Simonoff E, Treasure J, Field AE. Adolescent eating disorder behaviours and cognitions: gender-specific effects of child, maternal and family risk factors. *Br J Psychiatry* 2015; **207**: 320–7.

5 Schellong K, Schulz S, Harder T, Plagemann A. Birth weight and long-term overweight risk: systematic review and a meta-analysis including 643,902 persons from 66 studies and 26 countries globally. *PLoS One* 2012; **7**: e47776.

6 Yu Z, Han S, Zhu J, Sun X, Ji C, Guo X. Pre-pregnancy body mass index in relation to infant birth weight and offspring overweight/obesity: a systematic review and meta-analysis. *PLoS One* 2013; **8**: e61627.

7 Su Y, D'Arcy C, Meng X. Research Review: Developmental origins of depression – a systematic review and meta-analysis. *Journal of Child Psychology and Psychiatry* 2021; **62**: 1050–66.

8 Dachew BA, Ayano G, Betts K, Alati R. The impact of pre-pregnancy BMI on maternal depressive and anxiety symptoms during pregnancy and the postpartum period: A systematic review and meta-analysis. *J Affect Disord* 2021; **281**: 321–30.

9 Goodman A, Heshmati A, Malki N, Koupil I. Associations Between Birth Characteristics and Eating Disorders Across the Life Course: Findings From 2 Million Males and Females Born in Sweden, 1975-1998. *American Journal of Epidemiology* 2014; **179**: 852–63.

10 Micali N, Daniel RM, Ploubidis GB, De Stavola BL. Maternal Prepregnancy Weight Status and Adolescent Eating Disorder Behaviors: A Longitudinal Study of Risk Pathways. *Epidemiology* 2018; **29**: 579–89.

11 Taylor AE, Carslake D, de Mola CL, *et al.* Maternal Smoking in Pregnancy and Offspring Depression: a cross cohort and negative control study. *Sci Rep* 2017; **7**: 12579.

12 Kessler RC, Andrews G, Colpe LJ, *et al.* Short screening scales to monitor population prevalences and trends in non-specific psychological distress. *Psychological Medicine* 2002; **32**: 959–76.

13 Condon JT, Corkindale CJ. The assessment of parent-to-infant attachment: Development of a self-report questionnaire instrument. *Journal of Reproductive and Infant Psychology* 1998; **16**: 57–76.

14 Lampard AM, Franckle RL, Davison KK. Maternal depression and childhood obesity: A systematic review. *Preventive Medicine* 2014; **59**: 60–7.

15 Pearson RM, Evans J, Kounali D, *et al.* Maternal Depression During Pregnancy and the Postnatal Period: Risks and Possible Mechanisms for Offspring Depression at Age 18 Years. *JAMA Psychiatry* 2013; **70**: 1312–9.

16 Gander M, Sevecke K, Buchheim A. Eating disorders in adolescence: attachment issues from a developmental perspective. *Front Psychol* 2015; **6**: 1136.

17 Anderson SE, He X, Schoppe-Sullivan S, Must A. Externalizing behavior in early childhood and body mass index from age 2 to 12 years: longitudinal analyses of a prospective cohort study. *BMC Pediatr* 2010; **10**: 49.

18 Henderson M, Bould H, Flouri E, *et al.* Association of Emotion Regulation Trajectories in Childhood With Anorexia Nervosa and Atypical Anorexia Nervosa in Early Adolescence. *JAMA Psychiatry* 2021; **78**: 1249–57.

19 Srinivasan R, Flouri E, Lewis G, Solmi F, Stringaris A, Lewis G. Changes in Early Childhood Irritability and Its Association With Depressive Symptoms and Self-Harm During Adolescence in a Nationally Representative United Kingdom Birth Cohort. *Journal of the American Academy of Child & Adolescent Psychiatry* 2023; **0**. DOI:10.1016/j.jaac.2023.05.027.

20 Hogan AE, Scott KG, Bauer CR. The Adaptive Social Behavior Inventory (ASBI): A new assessment of social competence in high-risk three-year-olds. *Journal of Psychoeducational Assessment* 1992; **10**: 230–9.

21 Zhou N, Liang Y, Cao H, Chen Y, Lin X, Zhang J. Body mass index and internalizing symptoms from early childhood through early adolescence: Trend of codevelopment and directionality. *J Child Psychol Psychiatry* 2022; **63**: 324–32.

22 Patalay P, Sharpe H, Wolpert M. Internalising symptoms and body dissatisfaction: untangling temporal precedence using cross-lagged models in two cohorts. *J Child Psychol Psychiatry* 2015; **56**: 1223–30.

23 Gill D, Brewer CF, Del Greco M F, *et al.* Age at menarche and adult body mass index: a Mendelian randomization study. *Int J Obes* 2018; **42**: 1574–81.

24 Sequeira M-E, Lewis SJ, Bonilla C, Smith GD, Joinson C. Association of timing of menarche with depressive symptoms and depression in adolescence: Mendelian randomisation study. *Br J Psychiatry* 2017; **210**: 39–46.

25 McNicholas F, Dooley B, McNamara N, Lennon R. The Impact of Self-Reported Pubertal Status and Pubertal Timing on Disordered Eating in Irish Adolescents. *European Eating Disorders Review* 2012; **20**: 355–62.

26 Anderson SE, Sacker A, Whitaker RC, Kelly Y. Self-regulation and household routines at age three and obesity at age eleven: longitudinal analysis of the UK Millennium Cohort Study. *Int J Obes (Lond)* 2017; **41**: 1459–66.

27 O'Loughlin J, Casanova F, Fairhurst-Hunter Z, *et al.* Mendelian randomisation study of body composition and depression in people of East Asian ancestry highlights potential setting-specific causality. *BMC Med* 2023; **21**: 37.

28 Dion J, Blackburn M-E, Auclair J, *et al.* Development and aetiology of body dissatisfaction in adolescent boys and girls. *Int J Adolesc Youth* 2015; **20**: 151–66.

29 Sutaria S, Devakumar D, Yasuda SS, Das S, Saxena S. Is obesity associated with depression in children? Systematic review and meta-analysis. *Archives of Disease in Childhood* 2019; **104**: 64–74.

30 Bornioli A, Lewis-Smith H, Slater A, Bray I. Body dissatisfaction predicts the onset of depression among adolescent females and males: a prospective study. *J Epidemiol Community Health* 2020; : jech-2019-213033.

31 Liu C-Y, Schoeler T, Davies NM, *et al.* Are there causal relationships between attention-deficit/hyperactivity disorder and body mass index? Evidence from multiple genetically informed designs. *International Journal of Epidemiology* 2021; **50**: 496–509.

32 Haynes A, Kersbergen I, Sutin A, Daly M, Robinson E. A systematic review of the relationship between weight status perceptions and weight loss attempts, strategies, behaviours and outcomes. *Obes Rev* 2018; **19**: 347–63.

33 VanderWeele TJ. Principles of confounder selection. *Eur J Epidemiol* 2019; **34**: 211–9.

34 Van Roy B, Groholt B, Heyerdahl S, Clench-Aas J. Understanding discrepancies in parent-child reporting of emotional and behavioural problems: Effects of relational and socio-demographic factors. *BMC Psychiatry* 2010; **10**: 56.

35 Daniel RM, De Stavola BL, Cousens SN. Gformula: Estimating Causal Effects in the Presence of Time-Varying Confounding or Mediation using the G-Computation Formula. *The Stata Journal*; **11**: 479–517.
